# Randomized comparison of vaginal self-sampling by standard vs. dry swabs for Human papillomavirus testing

**DOI:** 10.1186/1471-2407-13-353

**Published:** 2013-07-22

**Authors:** Isabelle Eperon, Pierre Vassilakos, Isabelle Navarria, Pierre-Alain Menoud, Aude Gauthier, Jean-Claude Pache, Michel Boulvain, Sarah Untiet, Patrick Petignat

**Affiliations:** 1 Department of Gynecology and Obstetrics, Geneva University Hospitals and Faculty of Medicine, Boulevard de la Cluse 30, 1211, GENEVA 14, Switzerland; 2 Geneva Foundation for Medical Education and Research, Geneva, Switzerland; 3 Unilabs SA, Molecular Diagnostics Unit, Lausanne, Switzerland; 4 Department of Genetic and Laboratory Medicine, University Hospitals of Geneva and Faculty of Medicine, Geneva, Switzerland

**Keywords:** Cervical cancer screening, HPV, Human papillomavirus, Self-collected, Self-HPV, Self-sampling

## Abstract

**Background:**

To evaluate if human papillomavirus (HPV) self-sampling (Self-HPV) using a dry vaginal swab is a valid alternative for HPV testing.

**Methods:**

Women attending colposcopy clinic were recruited to collect two consecutive Self-HPV samples: a Self-HPV using a dry swab (S-DRY) and a Self-HPV using a standard wet transport medium (S-WET). These samples were analyzed for HPV using real time PCR (Roche Cobas). Participants were randomized to determine the order of the tests. Questionnaires assessing preferences and acceptability for both tests were conducted. Subsequently, women were invited for colposcopic examination; a physician collected a cervical sample (physician-sampling) with a broom-type device and placed it into a liquid-based cytology medium. Specimens were then processed for the production of cytology slides and a Hybrid Capture HPV DNA test (Qiagen) was performed from the residual liquid. Biopsies were performed if indicated. Unweighted kappa statistics (к) and McNemar tests were used to measure the agreement among the sampling methods.

**Results:**

A total of 120 women were randomized. Overall HPV prevalence was 68.7% (95% Confidence Interval (CI) 59.3–77.2) by S-WET, 54.4% (95% CI 44.8–63.9) by S-DRY and 53.8% (95% CI 43.8–63.7) by HC. Among paired samples (S-WET and S-DRY), the overall agreement was good (85.7%; 95% CI 77.8–91.6) and the κ was substantial (0.70; 95% CI 0.57-0.70). The proportion of positive type-specific HPV agreement was also good (77.3%; 95% CI 68.2-84.9). No differences in sensitivity for cervical intraepithelial neoplasia grade one (CIN1) or worse between the two Self-HPV tests were observed. Women reported the two Self-HPV tests as highly acceptable.

**Conclusion:**

Self-HPV using dry swab transfer does not appear to compromise specimen integrity. Further study in a large screening population is needed.

**Trial registration:**

ClinicalTrials.gov: NCT01316120

## Background

Cervical cancer incidence and mortality have decreased considerably since the introduction of cervical cancer screening programs in Western countries. However, despite these advances in secondary prevention, there are 500,000 new cases every year worldwide, mostly (85%) in developing countries [1-3]. Cervical cancer is predominantly a disease of low-resource countries because of limited access to healthcare and lack of cervical cancer screening programs [4]. Current data indicate that testing for high-risk human papillomavirus (HPV) types could be used as a primary screening method, and allowing women to do the sampling by themselves (Self-HPV) has been shown to have results similar to those obtained by health care professionals [5-8]. In countries with an existing cervical cancer screening program, Self-HPV is regarded as a possible alternative for women who decline to participate in the existing screening programms [9,10]. Many developing countries have limited or no screening resources, due to the prohibitively high cost of cytology-based screening and lack of qualified health care professionals. Self-HPV has the potential to overcome some of these barriers [4-11]. Available data regarding Self-HPV studies have been generated from samples collected with standard “wet” transport media like phosphate-buffered saline (PBS) or other transport media developed more recently. PBS is inexpensive but requires refrigeration, while newer transport media can be stored at room temperature but are costlier.

Acceptability studies for Self-HPV indicate that the method is generally well accepted by women, but revealed that some of women have doubts about the validity of the method. One of these was the concern about manipulating the test tube and spilling out the transport medium during the sampling procedure, which some patients interpreted as incorrect and feared that it might affect the test result [12,13]. This is an important issue, because it might lead to lower acceptability and participation rates in screening programs using Self-HPV. Finally, for low resource settings, a standard transport medium may be impractical and unavailable, because of the cost. Dry vaginal swabs may be more convenient and less expensive. Small studies suggest that HPV tests sampled by physicians using dry vaginal swabs are as accurate as those performed with standard transport medium for HPV detection [12,14,15]. The feasibility of Self-HPV with dry swabs transported and stored at room temperature might facilitate screening strategies in low-resource settings. To address this question, the aim of our study was to assess the performance of Self-HPV using dry swabs (S-DRY) compared with Self-HPV using wet transport medium (S-WET). We also explored the acceptability of the two Self-HPV methods.

## Methods

This study was conducted by the Geneva University Hospitals, Switzerland. The Ethics Committee of the Geneva University Hospitals, Switzerland, approved the study (number of approval: CE 10–184 MAT-PED 10–044). A signed informed consent form (ICF) was required for enrollment of participants in the study. This trial was registered at ClinicalTrials.gov (Identifier: NCT01316120).

### Patients

A total of 120 women were prospectively enrolled from our colposcopy clinic between November 2010 and August 2011. We randomized the sequence of the two HPV tests to avoid any potential biases that may advantage the first test. We included women aged 20 years or older, who understood the study procedures and accepted to participate by signing the ICF. Exclusion criteria were pregnancy and previous conization or hysterectomy.

### Procedures

The participants were randomized and received oral instructions by a physician or a research nurse about how to perform the Self-HPV, a self-collected vaginal sample. In brief, they were instructed to wash their hands before performing the procedure, to insert the swab into the vagina and to rotate it three times in both directions. The women were handed two self-sampling kits (S-DRY: a Dacron swab with a plastic bag; S-WET: a flocked swab with a tube filled with 1 ml of liquid transport medium (ESwab®, Copan, Brescia, Italy)) and were directed to a private, well-lit room to perform both samplings. Subsequently the participants were asked to complete a questionnaire on demographic characteristics, knowledge about HPV and preference between the two Self-HPV methods. The questionnaire used a 4-point scale to measure the degree of acceptability, physical discomfort and pain felt using the Self-HPV as well as the preference between the two Self-HPV methods. Participants then underwent a colposcopic examination. During this procedure, a cervical sample for liquid-based cytology and Hybrid Capture (HC) HPV test(QIAGEN AG Garstligweg 8 CH-8634 Hombrechtikon, Switzerland) was obtained using a broom type cervical brush and rinsing it in Preservcyt™ buffer solution (Hologic, Inc Bedford, Massachusetts, U.S.). A biopsy for histological analysis was performed if necessary.

Self samples were stored at room temperature and the time between sample collection and analysis ranged between 5 to 15 days.

### HPV testing

#### Real time PCR

Material from dry swabs (S-Dry samples) was placed into 1 ml of sterile phosphate-buffered saline (PBS)and the tubes were vortexed for 3×15 sec. Then, 0.5 ml of each sample was used for nucleic acid extraction and the rest was frozen at −20°C for storage. Tubes containing S-Wet samples (1 ml) were also vortexed for 3×15 sec and then, 0.5 ml of each sample was used for nucleic acid extraction and the rest of sample was frozen at −20°C for storage. HPV extraction, detection and genotyping were carried out from S-Dry and S-Wet specimens using the Cobas 4800 *(Roche Diagnostics International Ltd Forrenstrasse 2 CH-6343 Rotkreuz, Switzerland)* HPV test according to the manufacturer’s recommendations. This assay is an automated DNA extraction, PCR amplification and real-time detection of 14 High Risk HPV (HR-HPV) genotypes. It uses the beta-globin gene as an internal extraction and amplification control. PCR amplification and detection are performed in a single tube, where probes with four different reporter dyes track the different targets in the multiplex reaction: (i) HPV 16, and 18 individually, (ii) 12 HR-HPV types (i.e., HPV 31, -33, -35, -39, -45, -51, -52, -56, -58, -59, -66, and −68) as a group, and (iii) beta-globin.

#### Hybrid Capture (HC2)

Postcytology vials processed on the ThinPrep 2000 System ((Hologic,Inc Bedford, Massachusetts, U.S.) were used. At least 4 mL of remaining PreservCyt™ solution was used for the Hybrid Capture HPV DNA test. Samples were processed in the sample conversion kit and tested with HC2 according to the manufacturer's protocol with probe B for HR genotypes (a pool of full length HPV RNA probes against 13 HR-HPV genotypes including types 16, 18, 31, 33, 35, 39, 45, 51, 52, 56, 58, 59 and 68). Sample reactivity was measured in relative light units (RLU). A specimen was considered positive for HR HPV DNA if the ratio of the specimen RLU to the mean RLU of triplicates of a positive control at 1 pg per ml was >3.00. Samples with ratios between 1.00 and 3.00 were retested twice and were considered positive if 2 out of 3 results had a ratio >1.00.

Both methods used in this study, (HC and real time PCR) are FDA approved diagnostic methods. Because HC is considered as the reference standard to analyze physician-sampled specimen, as any methods in consideration to replace it should be as accurate as this method of screening.

#### Statistical analysis

The sample size necessary to validate a difference in test performance of 10% or more was calculated assuming a HPV prevalence of at least 40%. The proportion of positive agreement (PPA) between paired S-WET and S-DRY samples was calculated by dividing the number of samples testing positive for HPV in both tests by the number of samples testing positive in either S-WET or S-DRY. Cohens Kappa was calculated to measure the inter-test agreement between the self-sampling methods in terms of HPV risk categories. Positive agreement between S-WET and S-DRY was calculated as described by Wolfrum et al. [16].

## Results

One hundred twenty women were included in the study, of whom we excluded four for not having paired samples, three for inconclusive HPV test results (all three were S-WET samples) and one who refused further participation after performing Self-HPV. For the study analysis we included 112 women with 224 paired samples and completed questionnaires. The median age of participants was 31 years (range 21–63 years).

The HPV prevalence was 68.7% (95% Confidence Interval (CI) 59.3–77.2) detected by S-WET, 54.4% (95% CI 44.8–63.9) by S-DRY and 53.8% (95% CI 43.8–63.7) by HC. Mono-infections with HPV 16 or 18 were identified in 19.4% of participants, and combined infections with HPV 16 or 18 and other high-risk types were identified in 36.3%. Infection with one or more HPV types other than HPV 16 or 18 was observed in 44.1% of participants. HPV 16 was detected in 49.3% of cases and HPV 18 in 9%.

**Figure 1:**
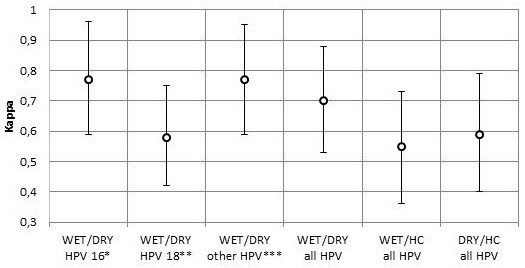
**Agreement of type-specific HPV detection between S-WET, S-DRY and HC.** Note: S-DRY = dry vaginal swabs used for self-sampling; S-WET = vaginal swabs with wet transport medium used for self- sampling; HC = Hybrid Capture physician sampeld; *HPV 16 in single or mixed infections; **HPV 18 in single or mixed infections; ***One or more of the non-16/18 high risk HPV types, in single or mixed infections.

The overall test agreement between S-WET and S-DRY was 85.7% (95% CI 77.8–91.6), with a 79.2% positive agreement. Cohen’s kappa for inter-test agreement was 0.70 (95% CI 0.53-0.88). Positive agreement for type specific HPV was 77.3% (95% CI 68.2–84.9). The inter-test agreement was good between S-WET and S-DRY for type-specific detection of HPV 16 and non-16/18 HPV types as well as for all HPV positive cases. The inter-test agreements between HC and S-DRY and between HC and S-WET were inferior to the inter-test agreement of S-WET and S-DRY (Figure 1, Table 1).

**Table 1:** Pooled data on women testing positive for HPV with S-WET, S-DRY and HC (Physician-sampling) by the grade of cytology (N=104) and histopathology (N=73)

**Grade of cytology**	**Total (N)**	**S-WET % (95% CI)**	**S-DRY % (95% CI)**	**HC % (95% CI)**
NILM	27	55.6 (35.3-74.5)	33.3 (16.5-54.0)	40.7 (22.4-61.2)
ASC-US*	35	60.0 (42.1-76.1)	48.6 (31.2-66.1)	48.6 (31.2-66.1)
LSIL	33	78.8 (61.1-91.0)	69.7 (51.3-84.4)	63.6 (45.1-79.6)
HSIL	9	100 (66.4-100)	88.9 (51.8-99.7)	66.7 (29.9-92.5)
Grade of histology				
Normal	28	53.8 (33.9-72.5)	39.3 (21.5-59.4)	28.6 (13.2-48.7)
CIN 1	22	72.7 (49.8-89.3)	68.2 (45.1-86.1)	72.7 (49.8-89.3)
CIN 2+	23	91.3 (72.0-98.9)	73.9 (51.6-89.8)	69.6 (47.1-86.8)

Note: *S-DRY* dry vaginal swabs used for self-sampling, *S-WET* vaginal swabs with wet transport medium used for self-sampling, *HC* Hybrid Capture, *NILM* Negative for intraepithelial lesion or malignancy: *ASC-US* Atypical squamous cells of undetermined significance, *ASC-H* Atypical squamous cells-cannot rule out high grade, *AGC* Atypical glandular cells, *LSIL* Low-grade squamous intraepithelial lesion, *HSIL* High-grade squamous intraepithelial lesion, *CIN* Cervical intraepithelial neoplasia; *ASC-US summarizes ASC-US, ASC-H, AGC.

Cytological diagnosis was available for 111 cases. For the comparison of S-WET and S-DRY with HC we excluded seven cases that were missing HC results. Histological diagnosis was performed in 73 cases, in the other cases no biopsies were taken because of normal colposcopy (Table 2).

**Table 2:** Comparison of S-WET and S-DRY in identifying various HPV genotypes

**HPV detected**	**Number of samples**				Positive agreement*
	**WET+ DRY+**	**WET+ DRY-**	**WET- DRY+**	**WET- DRY-**	
Subject level comparison					
Any HPV	61	16	0	35	88.4%
HPV 16 and/or HPV 18	32	13	2	65	81.0%
Other HR-HPV	47	12	1	52	87.9%

Note: HPV prevalence analyzed on subject level: subjects infected with HR-HPV/ specific HR-HPV types compared with those not infected with HR-HPV/ specific HR-HPV types.

*The observed proportion positive agreement=2a/(N+(a2d)), where a=the number of samples that were positive for HPV in both the wet and dry samples, d=the number of samples that were negative for HPV in both the wet and dry samples and N=all samples tested for HPV.

*S-DRY* dry vaginal swabs used for self-sampling, *S-WET* vaginal swabs with wet transport medium used for self-sampling.

In cases of abnormal cytology, the discordance of HPV results was at a consistent level of about 10% for all stages of severity. The discordance was elevated at 22.5% in cases of normal cytology. HPV positivity increased with increasing severity of cytological diagnosis, and reached 100% in HSIL (using S-WET). No difference in sensitivity or specificity was found between S-WET and S-DRY.

In the case of CIN 1 or higher lesions, the stratified overall test agreement was 88.6% (κ=0.70). S-DRY detected HPV in 73.9% of cases of CIN2/3, while S-WET was positive in 91.3% and HC was positive in 69.6%. The agreement between S-WET and S-DRY was highest in CIN1. The performance of both self-tests was comparable to HC samples taken by a physician.

We did not observe any statistically significant difference between S-DRY and HC or between S-WET and HC (Table 2).

One hundred and twelve questionnaires were completed, and no difference in acceptability between the two Self-HPV tests was observed. Most women (96.4%) were favorable to the idea of performing self-sampling at home, while three women (2.7%) were opposed to this idea and one did not answer the question. Fifty women experienced Self-HPV as slightly to severely painful or embarrassing, but no preference could be highlighted between the two Self-HPV tests. Some of the women (15/112 for S-WET; 6/112 for S-DRY) evaluated one of the self-tests as more complex than the other, while the majority did not report any difference in complexity. Twenty-seven women had higher confidence in S-WET, while two had higher confidence in S-DRY, but the large majority (83/112) thought both self-tests were equally reliable.

## Discussion

In recent years, we have learned that, with appropriate instruction, self-sampling of HPV specimens yield similar results compared with those collected by health care professionals. The high percentage of agreement for high-risk HPV between these two approaches suggests that Self-HPV is a promising alternative to physician-sampling for HPV testing and cervical cancer screening [17]. Belinson et al. reported that self-collected vaginal specimens showed lower sensitivity and lower specificity than physician collected endocervical specimen analyzed with HC, but they showed that self-collected samples using more sensitive PCR-based assays could improve the sensitivity to a comparable level [18,19].

Recent studies reported that Self-HPV testing is more sensitive than the Pap test in detecting CIN2+ in Chineese and Mexican women of low socioeconomic status [8,20]. Furthermore, a review by Gravitt, et al. includes the performance of self- vs clinician collected swabs for detection of CIN2+ which provides a better assessment of alternatives to physician sampling for cervical cancer screening [21].

The possibility of using Self-HPV stored at ambient temperature without transport media would clearly enhance and simplify the utility of Self-HPV. This could reduce the costs of the methods, which might be attractive for low-resource countries. Moreover, it may reduce women’s concerns that the test quality is reduced by accidentally spilling out some of the transport medium [12,13]. We found that swabs transported in a dry state were as accurate as those obtained with swabs shipped in a wet transport medium, in terms of quality of results. Of note, all dry samples were sufficient for analysis while three of the wet samples were insufficient. The test performance between S-WET and S-DRY methods was similar according to the overlap of the 95% confidence intervals. Cohen’s kappa calculated for the inter-test agreement (0.7) corresponds to a substantial agreement and is in line with the results of other studies addressing this question in the context of physician-sampling [14,15,22]. Shah et al. compared vaginal swabs performed by physicians, with and without transport medium, and established kappa values ranging between 0.69 and 0.81 [15]. Likewise, similar to our results, the concordance of the S-DRY and S-WET results was higher compared with the concordance of any Self-HPV, wet or dry, with the physician-sampled specimen [15]. If S-WET is used as a screening method, women should be reassured about the good test performance to ensure trust in the screening results.

In this study we used different swabs for S-WET and S-DRY. The S-WET swab was a flocked swab that consists of fine and short filaments fixed at the top of the stick while the swab used the S-DRY swab was a standard Dacron swab consisting of a long filament enrolled around a stick. Krech et al. compared flocked and rayon swabs for their sensitivity to detect HPV infection and noticed that the sensitivity of flocked swabs was higher [12]. They explained this observation with a higher capacity of adhesion in the flocked swabs, which leads to a better proportion of DNA detection [12]. This devices difference might explain the trend of our results in favor of S-WET. However, the use of different swabs did not cause significant differences in test performance, which might even give room to the question if a simple Dacron swab used without transport medium might be an acceptable alternative.

A shortcoming of our study is the use of different HPV DNA detection assays for self-collected vaginal specimens and physician-collected endocervical specimens. The number of variables that differ in addition to wet vs. dry transport weakens our conclusion about the feasibility of dry transportation. Other weaknesses of our study are the small sample size and the fact that our population was recruited in a colposcopic clinic having a high rate of HPV prevalence. Even though the HPV positivity in S-WET compared with S-DRY is not statistically significant, it seems to point in the direction of S-DRY being slightly less sensitive than S-WET. Additional work is needed to conduct a study in a real context of screening with a larger sample size to determine if S-DRY and S-WET have equal sensitivity.

## Conclusions

Our study shows that Self-HPV swabs can be successfully transported in a dry state at ambient temperature without greatly altering specimen integrity. Self-sampling for HPV testing using S-DRY could be an alternative to other self-sampling methods that require “wet” transport media. Advantages of the dry method include a simplification of the method , as well as its ease of use and lower cost may be of advantage for women with restricted access to health care delivery. However, further research is needed to confirm the equality of both methods in a large screening population.

## Abbreviations

ASC-US: Atypical squamous cells of undetermined significance; AUC: Area under the curve; CIN 1: Cervical intraepithelial neoplasia grade 1; CIN 2/3: Cervical intraepithelial neoplasia grade 2 or 3; HC: Hybrid Capture; HPV: Human Papillomavirus; HSIL: High-grade squamous intraepithelial lesion; ICF: Informed consent form; LSIL: Low-grade squamous intraepithelial lesion; NPV: Negative predictive value; PBS: Phosphate-buffered saline; physician-sampling: Physician collected cervical sample for HPV testing; PPA: Proportion of positive agreement; PPV: Positive predictive value; ROC curve: Receiver Operating Characteristic curves; S-DRY: Self-HPV using a dry swab; S-WET: Self-HPV using a standard wet transport medium; Self-HPV: Self-sampling for HPV testing using a vaginal swab.

## Competing interests

The authors declare that they have no competing interests.

## Authors’ contributions

IE conducted the study and drafted the manuscript. VP designed the study. IN participated in coordinating the study. PAM and GA were responsible for the molecular biological analysis. JCP co-designed the study. MB directed the statistical analyses. SU conducted statistical analyses and contributed to the manuscript. PP co-designed the study and led the research project. All authors read and approved the final manuscript.

## Pre-publication history

The pre-publication history for this paper can be accessed here:

http://www.biomedcentral.com/1471-2407/13/353/prepub
